# Impact of Physiotherapy Intervention on Pain, Quality of Life, and Function in Low Back Pain Associated With Piriformis Syndrome: Protocol for Systematic Review

**DOI:** 10.2196/72350

**Published:** 2026-03-24

**Authors:** Nikita Deshmukh

**Affiliations:** 1 Ravi Nair Physiotherapy College, Datta Meghe Institute of Higher Education and Research, Sawangi (Meghe), Wardha, Maharashtra, 442001, India, 91 09561950640

**Keywords:** neuromuscular condition, back discomfort, misdiagnosed, affected quality of life, low back pain, piriformis, sciatica, buttock pain, physiotherapy intervention

## Abstract

**Background:**

Piriformis syndrome is a neuromuscular condition with hip and buttock pain and other symptoms, including referred pain towards the lower back and leg and radiating towards the foot’s medial aspect. Similarly, low back pain caused by piriformis syndrome is undetected or difficult to diagnose because of similar symptoms of lumbar disc herniation, lumbar stenosis, or radiculopathy, as well as neurogenic pain. A study conducted in 2013 found 2910 patients experienced low back pain with sciatica, which is the most common cause of low back pain, because of piriformis muscle stiffness. The prevalence of low back pain in piriformis syndrome is 5%‐36%. It is more commonly seen in women than men.

**Objective:**

This systematic review protocol seeks to identify evidence whether physiotherapy interventions effectively relieve pain, improve functional outcomes, and enhance quality of life among individuals experiencing low back pain associated with piriformis syndrome.

**Methods:**

This review will analyze randomized controlled trials (RCTs) that include physiotherapy for patients with low back pain linked to piriformis syndrome. The included studies must report on pain levels or improvements in function related to quality-of-life outcomes. Searches will take place using Google Scholar, Pubmed/MEDLINE, the Cochrane Library, and PEDro for articles published from January 2014 to January 2025. Two reviewers will individually check the studies, choose relevant ones, and collect data while assessing quality using the Cochrane risk of bias tool. We will provide a narrative summary of the findings, concentrating on data about pain management, functional improvement, and quality of life enhancements.

**Results:**

This review will synthesize knowledge focusing on pain, quality of life and functions in low back pain, which is associated with piriformis syndrome. A synthesis of the findings will be conducted to determine which components of the interventions identified were the most advantageous to the patient population.

**Conclusions:**

The systematic review protocol is designed to identify the effectiveness of physiotherapy interventions for managing low back pain in piriformis syndrome. This analysis will review RCTs with evidence-based recommendations on reducing pain, improving function, and enhancing quality of life.

## Introduction

Piriformis syndrome (PS) is a neuromuscular condition characterized by the cardinal symptoms. Sometimes, the syndrome includes hip and buttock pain. The pain radiates towards the back of the leg, sometimes referred down to the medial aspect of the foot. Although this peripheral neuritis is clinically indistinguishable from L5 or S1 radiculopathy, it secondarily leads to abnormal piriformis muscle actions. This abnormal muscle activity results in the compression and irritation of the sciatic nerve as it courses under or through the piriformis muscle [1]. The associated symptoms are challenging to diagnose due to their similar presentation to lumbar disc herniation with radiculopathy, neurogenic pain, and stenosis with radiculopathy. A study showed that in 2013, approximately 2910 patients suffered from low back or buttock pain with sciatica, and 183 (6.25%) patients were diagnosed with PS. If the diagnosis of PS is delayed, it may lead to pathological complications such as chronic somatic dysfunction and sciatic nerve compression; additionally, compensatory changes may occur, leading to muscle weakness, paraesthesia, hyperesthesia and pain [1,2].

Patients experience discomfort in the back of the thigh, hip area, and lower back, spreading to the leg and foot. Key signs of this condition include numbness, a burning feeling, trouble walking, and difficulty in carrying out everyday tasks. The incidence of this condition ranges from 5% to 36% among individuals with low back pain (LBP). Piriformis syndrome is often misdiagnosed or overlooked in clinical settings due to its symptom overlap with additional ailments, including sacroiliac joint dysfunction, intervertebral disc herniation, and lumbar radiculopathy. Without appropriate treatment, the condition can lead to significant difficulties with prolonged sitting, walking, and standing [3,4]. Piriformis syndrome is a chronic mechanical dysfunction that arises when prolonged sitting places continuous stress on the belly of the piriformis muscle. This leads to muscle spasm and inflammation, resulting in compression of the sciatic nerve. Activities like walking or sitting with one leg crossed can worsen symptoms. Chronic overload, overuse fatigue, radiating limb pain, and severe injuries that may activate skeletal muscle trigger points are additional contributing causes. Sacroiliac joint dysfunction can also result in trigger points in the piriformis muscle, further hindering proper muscle activity. Sacroiliitis, LBP symptoms, or other hip pathologies can also exacerbate the condition [5-7].

Low back pain can often be prevented by encouraging patients to adopt proper sitting postures and avoid prolonged sitting. Various therapeutic approaches are effective in managing different types of LBP. These include manual treatment, electrical stimulation, traction therapy, bed rest, the use of assistive equipment, and heat. Usually, these techniques are regarded as the initial course of treatment for LBP [8]. People who have cerebral palsy, trauma, overexertion, a constricted sciatic foramen, and biomechanical abnormalities may be at risk for PS. These factors can subject the piriformis muscle to excessive stretching or shortening, increasing the risk of developing the condition [9,10]. LBP is among the most prevalent conditions in dancers, accounting for up to 25% of all dance-related injuries. Another study indicated that PS is most commonly observed in individuals in their 40s and 50s. It is more frequent in women than men due to biomechanical differences, such as the wider angle of the quadriceps femoris muscle associated with the female hip structure [11,12].

Tightness in the piriformis limits the range of motion and decreases flexibility, which affects a person’s ability to perform both physically and socially. If not identified early, it can progress to PS. Because its symptoms frequently resemble those of disorders including radiculopathy, sacroiliac joint dysfunction, intervertebral disc herniation, or sacroiliitis, misdiagnosis is frequent. However, piriformis tightness is also common in people with low back discomfort. Stiffness in the piriformis muscle is among the most common causes of LBP. Women have piriformis-related illnesses more frequently than men, most likely as a result of anatomical and biomechanical variations. Common signs and symptoms of PS include pain on sitting, muscle tenderness on palpation, muscle spasms, and pain during rectal examination. Piriformis syndrome is also a potential cause of sciatica-related low back and buttock pain. Piriformis syndrome should be considered in the differential diagnosis when pain travels from the back to the leg. Research indicates that most individuals with PS have a history of microtrauma, such as sitting on hard surfaces (36.9%), sitting cross-legged (10.8%), or prolonged walking or running (18.5%) [5,13].

Degenerative disc disease and spondylolysis are the most common causes of LBP in athletes. Diagnosing and managing LBP in athletes can be challenging, as pinpointing the exact source of pain often requires a thorough approach. Physicians must also consider fewer common causes of LBP in this population. While treatment typically targets identifiable causes, over 80% of LBP cases remain unexplained. It is challenging to detect unexplained LBP, which is frequently linked to lower-extremity nerve-related symptoms [14]. Low back pain is a multifactorial condition, requiring a thorough approach when conventional treatments fail to provide relief. A minimum diagnostic workup for athletic LBP should include a comprehensive history to rule out red-flag conditions, alongside specific, evidence-based imaging techniques. Because its definition and mechanism are still up for debate, PS, which is linked to LBP or buttock pain, is still a contentious diagnosis. PS is regarded as a trigger point-based fascial pain condition. Sitting for extended periods usually aggravates symptoms, such as sustained lower back flexion, simultaneous adduction, and internal rotation of the hip joint. These movements, particularly hip adduction and internal rotation in a low posture, are integral to athletic performance and can exacerbate symptoms in affected individuals [6,15]. Which physiotherapy interventions have the most substantial evidence for effectively managing LBP caused by PS? How do these interventions compare in their ability to reduce pain, improve functional limitations, and enhance quality of life for individuals with the LBP associated with PS?

## Methods

The eligibility criteria for this systematic review are based on the PICO principle and include the following:

- Exclusion criteria: Individuals who have prolapse intervertebral disc disease; sacro-iliac joint fracture, or posture-related LBP, or any postural impairment associated with LBP.
- Inclusion criteria:Population involves diagnosed cases of PS associated with LBP; dndividuals aged 26‐50 years, and male and female participants are involved; pain at the intersection of the sciatic nerve and the piriformis muscle; positive modified FAIR test and Lasegue’s manoeuvre test positive.
- Intervention: The review includes studies for PS-associated LBP in physical therapy intervention, such as: exercise therapy (ie, strengthening, stretching) [9]; manual therapy (ie, sacro-iliac joint mobilisations, active release technique, neuromuscular inhibition technique, post facilitation stretch technique) [16]; nerve flossing technique (NFT) [10]; postural corrections; appropriate foot support (ie, orthosis); and electrotherapy modalities (ie, shockwave therapy).
- Comparison: Studies must include a comparator group, which could be: positional release technique [1], myofascial release [3], post-isometric relaxation [16], tissue mobilization [16], or conventional physical therapy.
- Outcome measures: The following primary outcomes are used in the study at the beginning (preintervention and after the intervention): pain (using scales such as the Visual Analog Scale (VAS) or Numeric Rating Scale) [17]; functional improvement (Lower Extremity Functional Scale) [18]; and quality of life [19].
- Study design: The focus will be randomized controlled trials (RCTs).
- Language: Studies include only publications published in English because they are easy to understand.
- Publication date: Studies published from January 2014 to January 2025 will be considered.
- Publication type: Published studies like RCTs and relevant content.

### Information Sources

The literature search will encompass PubMed/MEDLINE, the Cochrane Library, and PEDro, focusing on English-language articles published between January 2014 and January 2025. Google Scholar will be used as a supplementary search engine.

### Search Strategy

A comprehensive literature search will be performed using PubMed/MEDLINE, the Cochrane Library, PEDro, and Google Scholar. The search strategy will include MeSH terms and relevant keywords shown in Textbox 1, such as PS.

The search will be updated before the final analysis to ensure the incorporation of the latest evidence.

Textbox 1. **MeSH terms and relevant keywords used in the search strategy.**

**PS:** “piriformis muscle syndrome”[MeSH Terms] OR (“piriformis”[All Fields] AND “muscle”[All Fields] AND “syndrome”[All Fields]) OR “piriformis muscle syndrome”[All Fields] OR (“piriformis”[All Fields] AND “syndrome”[All Fields]) OR “PS”[All Fields]. **PS:** “piriformis muscle syndrome”[MeSH Terms] OR (“piriformis”[All Fields] AND “muscle”[All Fields] AND “syndrome”[All Fields]) OR “piriformis muscle syndrome”[All Fields] OR (“piriformis”[All Fields] AND “syndrome”[All Fields]) OR “PS”[All Fields] **physical therapy**: “physical therapy modalities”[MeSH Terms] OR (“physical”[All Fields] AND “therapy”[All Fields] AND “modalities”[All Fields]) OR “physical therapy modalities”[All Fields] OR (“physical”[All Fields] AND “therapy”[All Fields]) OR “physical therapy”[All Fields]. **PS:** “piriformis muscle syndrome”[MeSH Terms] OR (“piriformis”[All Fields] AND “muscle”[All Fields] AND “syndrome”[All Fields]) OR “piriformis muscle syndrome”[All Fields] OR (“piriformis”[All Fields] AND “syndrome”[All Fields]) OR “PS”[All Fields] **Low back pain:** “low back pain”[MeSH Terms] OR (“low”[All Fields] AND “back”[All Fields] AND “pain”[All Fields]) OR “low back pain”[All Fields].

### Selection Process

The reviewers will independently extract data from the included studies using the established extraction form. Information on the study (purpose, sampling strategy, data source, recruitment period, intervention duration, and study duration), participant characteristics, interventions, comparisons, and results is included. The abstracts and titles will be screened for relevancy by two separate reviewers. The eligibility of pertinent full-text research publications will be evaluated next. Discussions or, if required, communication with a third reviewer will be used to resolve any disputes regarding research inclusion.

### Data Collection Process

A standardized extraction form, pilot-tested before full implementation, will be used by two independent reviewers to extract data. Essential data, such as research characteristics, participant demographics, intervention and comparator details, outcome measures, and findings, will be gathered via the form. Discussions or, if required, communication with a third reviewer will be used to resolve any disagreements or ambiguities in the data extraction [20].

### Data Items

Participant characteristics will include age (26‐50 y), gender distribution, inclusion and exclusion criteria, and baseline information on health, PS length, physical activity level, and other relevant clinical or demographic data. The sort of physiotherapy intervention employed will be covered in the intervention specifics (eg, exercise therapy (ie, strengthening, stretching); manual therapy (ie, sacroiliac joint mobilizations, active release technique, neuromuscular inhibition technique, post facilitation stretch technique; nerve flossing technique (NFT), postural corrections, appropriate foot support ie, orthosis), electrotherapy modalities (shockwave therapy), the duration, frequency, and intensity of the intervention and the mode of delivery (eg, home-based programs, group sessions, individual sessions). The kind of control or comparator interventions will be included in the comparator group (positional release technique, myofascial release, post isometric relaxation, tissue mobilization, conventional physical therapy), their duration, frequency, and intensity. Outcome measures will assess pain level (using methods likethe VAS or numeric rating Scale), improvement in function (measured with tools like the Lower Extremity Functional Scale), quality of life (36-Item Short Form Health Survey (SF-36)), and any side effects reported. Study details will cover study type (like randomized controlled trial), number of participants, funding sources to check for bias, and any conflicts of interest stated by authors. Planned data assumptions and simplifications will keep data extraction and analysis consistent and accurate across all studies.

### Outcomes

Ain intensity will be assessed using continuous scales such as the Visual Analogue Scale (VAS) or the Numeric Pain Rating Scale, with pre- and postintervention scores compared. Functional improvements will be assessed using lower-extremity function scale, which evaluates functional enhancements after treatment by comparing pre- and posttreatment scores. Standardized questionnaires or measures will be used to assess quality of life, such as the SF-36, which focuses on overall well-being, activities of daily living, and the social and emotional impact of well-being, with pre- and post-treatment assessments conducted (4 wks).

### Risk of Bias Assessment

The Cochrane Risk of Bias tool will assess the methodological quality and risk of bias in the included RCTs. The ROBINS-2 tool (Risk of Bias in Non-randomized Studies—of Interventions) will evaluate bias in observational research studies—new CASSTLE.

### Data Synthesis

The effectiveness of therapy for LBP linked to PS will be evaluated by calculating standardized mean differences with 95% CIs to measure primary outcomes such as pain intensity, quality of life, and functional improvement. A narrative approach will be the primary method used for data synthesis due to the anticipated variation among studies. A comprehensive summary of the results from the included studies will be provided, emphasizing the main conclusions, different kinds of therapies, and how they affect pain management, functional recovery, and quality of life. Subgroup analyses will be carried out to examine any differences in the effects of the intervention depending on participant demographics or intervention features.

### Confidence in Cumulative Evidence

The GRADE (Grading of Recommendations, Assessment, Development, and Evaluation) method will be applied in this systematic review to evaluate the quality of evidence regarding the effectiveness of physiotherapy interventions for LBP associated with PS. The GRADE framework will specifically be applied to the following outcomes: quality of life (measured with the SF-36), functional improvement (rated using the Lower Extremity Functional Scale), and pain severity (measured with the Visual Analog Scale or Numeric Rating Scale), based on comparisons of pre- and post-intervention scores.

## Results

**Figure 1.**
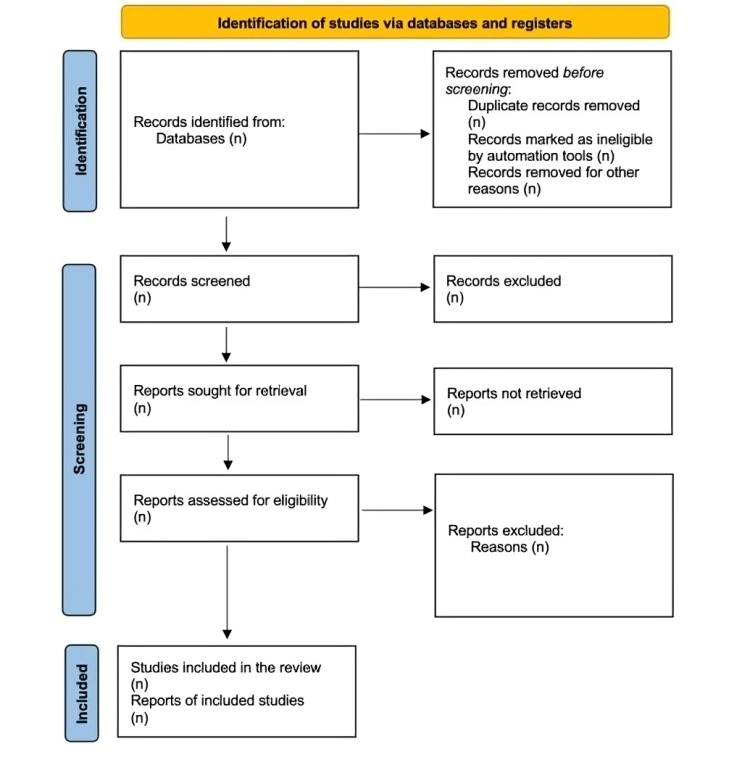
Preferred reporting items for systematic review and meta-analysis protocols (PRISMA-P) flowchart [20].

The purpose of this research is to determine if rehabilitative therapies are offered for LBP associated PS. The identified studies will be reported in a PRISMA flowchart (Figure 1) [20].

A synthesis of the results will take place to ascertain which elements of the indicated therapies were most beneficial to the patient group. The findings will be used to develop an evidence-based treatment plan to improve quality of life and aid future treatments. The review’s findings will be considered with the panel of experts and decision-makers to create a new evidence-based therapy plan for treating LBP in people with PS .

## Discussion

### Principal Findings

This systematic review protocol outlines a comprehensive approach to evaluating the effectiveness of various physiotherapy interventions for managing LBP associated with PS. PS, with a prevalence of 5%‐36%, significantly impacts quality of life and functional ability due to pain and limitations in daily activities. Underdiagnosis and inadequate treatment are common due to factors such as limited clinical knowledge, difficulty in differentiating PS from other causes of LBP, and a lack of appropriate physiotherapy management. Physical therapy interventions and other holistic approaches and evidence-based practices aim to address underlying factors such as muscular imbalances, biomechanical alterations, and pain to improve patient outcomes and prevent complications. Some evidence suggests promising effects of specific treatments for LBP associated with PS. The results of this systematic review will advance our knowledge and treatment of this illness by offering evidence-based suggestions to direct clinical practice, improve patient outcomes, influence evidence-based guidelines, and pinpoint areas for further study.

### Strengths and Limitations

The study addresses LBP associated with PS, a specific condition often overlooked in research, providing valuable insights. The review covers a holistic set of outcomes relevant to patient care by examining pain, quality of life, and function. The systematic review protocol ensures a structured and rigorous methodology, reducing bias and increasing the reliability of findings. The results can guide physiotherapists in developing targeted interventions and improving clinical practice. This review protocol can consolidate existing evidence on physiotherapy’s role in managing PS and address inconsistencies in the current literature. The limitations of physiotherapy interventions can vary widely, making comparisons and synthesis of results challenging. The availability of high-quality RCTs on LBP in PS may be limited, potentially affecting the robustness of the conclusions. Pain and quality of life are subjective and can vary between individuals, possibly introducing variability in the results. Studies with negative or inconclusive results may not be published, skewing the data toward positive outcomes. While focusing on PS is a strength, it may limit generalizability to other causes of LBP.

### Conclusion

This systematic review aims to evaluate the effectiveness of physiotherapy techniques for treating LBP associated with PS. The review will offer evidence-based suggestions to alleviate the pain, reduce functional limitations, and improve the quality of life for people with LBP in PS by examining RCTs. In addition to providing opportunities for future study to enhance the quality of life for those with LBP associated with PS, the findings will help inform clinical practice and enrich evidence-based physiotherapy.

## Supplementary material

10.2196/72350 Checklist 1 SPIRIT (Standard Protocol Items: Recommendations for Interventional Trials) checklist.

## Abbreviations

GRADE: Grading of Recommendations, Assessment, Development, and Evaluation
LBP: low back pain
PS: piriformis syndrome
RCT: randomized controlled trial
SF-36: 36-Item Short Form Health Survey
